# Definitions and scope of the mental health burden of global climate change

**DOI:** 10.1192/j.eurpsy.2023.1581

**Published:** 2023-07-19

**Authors:** F. Vergunst, R. Williamson, A. Mazzazza, H. Berry, M. Olff

**Affiliations:** 1 University of Oslo, Oslo, Norway; 2 University of Montana, Boseman, United States; 3 London School of Hygiene and Tropical Medicine, London, United Kingdom; 4 Macquarie University, Sydney, Australia; 5 Amsterdam University Medical Centre, Amsterdam, Netherlands

## Abstract

**Introduction:**

Climate change is increasing the frequency of extreme weather events – such as heatwaves, droughts, floods, and wildfires – and undermining the mental health and wellbeing of global populations, but the dimensions and scope of this burden remain under-studied.

**Objectives:**

To identify the distinct but overlapping mental health domains that are being impacted by climate change-related stressors and how these domains relate to and interact with one another.

**Methods:**

A narrative synthesis of conceptual and empirical studies of climate change and mental health.

**Results:**

We find strong empirical evidence that climate change is already harming mental health across multiple mental health domains, including through increased rates of psychiatric disorders (e.g., PTSD, depression, anxiety), sub-clinical psychological distress, harmful substance use, self-harm/suicidal behaviors, and worry about the observed and anticipated impacts of climate change. Most of the mental health burden is likely to occur in the form of sub-clinical symptoms, including lowered resilience and subjective well-being, while negative psychological states (e.g., eco-anxiety) are likely to constitute a smaller proportion of the overall burden. We argue that the mental health burden can be helpfully conceptualised within a dual-continuum model that considers the presence/absence of psychiatric diagnosis on the one hand, and high/low psychosocial wellbeing on the other.

**Image:**

**Figure:**
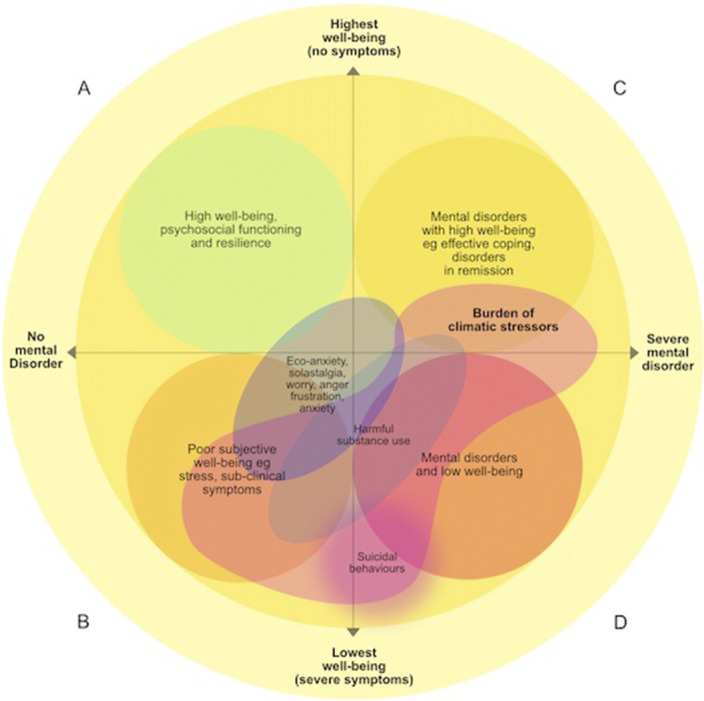

**Conclusions:**

Climate change is already harming the mental health of global populations across multiple functional domains. Defining and tracking the scope of this growing burden is essential so that effective preventive and adaptive action can be taken.

**Disclosure of Interest:**

None Declared

